# Leaf Biochemistry Parameters Estimation of Vegetation Using the Appropriate Inversion Strategy

**DOI:** 10.3389/fpls.2020.00533

**Published:** 2020-05-20

**Authors:** Lin Du, Jian Yang, Jia Sun, Shuo Shi, Wei Gong

**Affiliations:** ^1^School of Geography and Information Engineering, China University of Geosciences, Wuhan, China; ^2^Artificial Intelligence School, Wuchang University of Technology, Wuhan, China; ^3^State Key Laboratory of Information Engineering in Surveying, Mapping and Remote Sensing, Wuhan University, Wuhan, China; ^4^Collaborative Innovation Center of Geospatial Technology, Wuhan University, Wuhan, China

**Keywords:** spectral band correlation, artificial neural networks, band selection, vegetation biochemistry parameter, spectral property

## Abstract

Biochemistry parameters of vegetation are important indicators of the photosynthetic process and provide a substantial amount of data about the status of ecosystems. Estimation of these parameters are greatly affected by the correlations of spectral bands and the sensitivity of each biochemistry parameter to inversion models. Hence, reducing the spectral dimension and inefficient computation process using an appropriate inversion strategy is significant for biochemistry parameters’ estimation. In this work, we used band-selection-based artificial neural networks (ANNs) combined with feature weighting (FW) and principal component analysis (PCA) process to reduce the sensitive spectral correlations and to improve the inversion model predictability for four biochemistry parameters: chlorophyll a and b (Cab), carotenoid (Car), equivalent water thickness (EWT), and leaf mass per area (LMA). We analyzed the model performance by conducting different inversion strategies, including: (1) linking reflectance (R), transmittance (T), and R&T spectral properties in different numbers of band to four biochemistry parameters; (2) simultaneously and then separately inverting them using FW- and PCA-ANNs considering their sensitivity to the ANN model; and (3) choosing a spectral subset from R, T spectrum for EWT, and LMA inversion successively. The results show that: (i) the FW- and PCA-ANN models exhibit efficient improvements by selecting less spectral characteristics; (ii) concurrently inverting EWT and LMA can achieve a satisfactory *R*^2^, while it is inappropriate for Cab and Car whose optimal *R*^2^ are obtained by separately inverting all four biochemicals; (iii) the properties of R, T, and R&T spectra exhibit various performances on parameters inversion.

## Introduction

Vegetation is one of the most important components of the ecosystem on earth, and it assimilates CO_2_ while releasing O_2_ to maintain a normal energy exchange with the surrounding environment (Piao et al., 2006; Koetz et al., 2007). Meanwhile, the growing status of vegetation (GSV), including the health-stress status and the functioning process, refers to the proper functioning of the entire ecosystem (Féret et al., 2017). Biochemistry parameters [e.g., chlorophyll (Cab) and carotenoid (Car) contents, equivalent water thickness (EWT), leaf mass per area (LMA)], are significant indicators of photosynthesis activity, which is closely related with the GSV (Delegido et al., 2010). Therefore, accurate and fast estimation of them is a general but efficient approach to estimating the relationship between GSV and environmental stress.

Spectra of reflectance (R) and transmission (T) remotely sensed in wide range bands have been proven to be potential applications for biochemistry parameters’ estimation of vegetation (Sun et al., 2018). Several analysis methods about such spectral data, such as mathematics regression and intelligent algorithms, have been widely conducted. Most of these methods focused on linking spectral indices to Cab, EWT, and other pigments (Hunt and Rock, 1989; Delegido et al., 2010; Wu et al., 2010). Admittedly, it has indeed improved the analysis models by combining optimization algorithms, such as partial least squares (PLS) (Du et al., 2016), stepwise multiple linear regression (Liu et al., 2014), support vector machines (Mountrakis et al., 2011), and artificial neural networks (ANNs) (Latt and Wittenberg, 2014). However, some evident drawbacks, such as the used wavelengths not always being related with the compounds of interest but occasionally being associated with biomass, canopy structure, or other biochemicals, were observed (Yi et al., 2007). Moreover, the analysis model is inconsistent with vegetation types and even yields contrasting results because of different spectral properties, such as R, T, or R&T (Li et al., 2014; Sun et al., 2018). Furthermore, many uncertainties of the inversion model, such as the correlated and redundant information between bands and each biochemical, makes a great difference on the inversion results (Goel, 1988), but few pieces of research considered whether it was necessary to analyze these parameters simultaneously or separately. Therefore, appropriate selection of feature bands against special biochemicals should be conducted before applying numerous analysis models, including ANNs (Zhao et al., 2013). This is an efficient inversion strategy to increase the sensitivity of special spectral bands to each biochemical.

The ANNs can assimilate multidimension variables for relationship modeling of biochemicals by simulating human neurons. The workload of ANNs becomes considerably larger with substantial variables, and the effects of some correlated and redundant data from numerous bands and biochemicals will also be integrated into the models, thereby overwhelming most useful data of the compounds of interest. Consequently, the ANNs with high-dimension variables (both spectra and biochemicals) may inevitably suffer from the overlearning problem just like the overfitting in regression analysis (Yao et al., 2015). Yi et al. (2007) used principal component analysis (PCA) to select feature variables for ANNs for rice nitrogen status monitoring and then compared them using multiple linear regression. Although the authors obtained unsatisfactory *R*^2^ values, applying PCA-based models on the R spectrum showed great potential in nitrogen analysis. A study by Yang et al. (2017) has proven the availability of PCA-ANN on estimating the leaf nitrogen contents of rice based on laser-induced chlorophyll fluorescence LiDAR data. In the present study, another band selection method was utilized with the ANN model for comparison with PCA-ANNs, namely, feature weighting (FW), which was introduced by Huang and He (2005). FWs are calculated depending on the divergence between different classes, including vegetation species and biochemistry parameter contents. Thus, values of FWs directly relate sensitivity of feature bands to biochemicals. This is different from PCA whose selected variables, called principal components (PCs), are linear combinations of original data, thereby diminishing the data dimension without any loss of innate information about biochemistry parameters (Song et al., 2011).

This investigation aims to estimate the performance of inversion strategy in biochemistry parameter analysis using band-selection-based ANN models (FW- and PCA-ANNs). The analysis process was conducted by designing different strategies, including: (1) linking R, T, and R&T spectral properties in different numbers of bands to four biochemistry parameters; (2) simultaneously and then separately inverting them with the ANN method to evaluate whether analyzing all biochemicals together makes a great difference on model inversion; and (3) inverting the EWT and LMA in a spectral subset chosen from R, T spectrum successively.

## Databases and Analysis Methods

We firstly describe two experiment databases used in this study and then simulate R and T spectra using the PROSPECT-5 model (Féret et al., 2008) with special distribution features of the biochemical parameters. The range of synthetic parameters are limited based on two experimental databases. Different inversion strategies based on synthetic and experimental data are then compared to find the optimal one for each parameter. In the analysis process, the band-selection-based ANN models are used.

### Databases Description

Three databases were used in this study. Two of them were published independent databases, i.e., ANGERS and LOPEX. The first database was measured in Angers, France, by Jacquemoud et al. (in June 2003) (Féret et al., 2008). It contained 276 vegetation leaf samples (43 species) with their corresponding R and T spectra and a variety of biochemistry parameters, such as total Cab, Car, EWT, LMA, etc. The spectra of R and T (400–2450 nm) in this database were measured using the laboratory spectrophotometer or field spectroradiometers with different spectral resolutions. The details can be found in studies by Féret et al. (2008) and Lichtenthaler (1987). The second database (LOPEX) was measured by the Joint Research Center of European Commission in 1993 (Hosgood et al., 1995), which consisted of 320 samples, namely a total of 45 vegetation species, and the range of the wavelength was 400–2500 nm. In this database, five spectra were measured for each leaf, and only 64 fresh leaves were available. In both databases, we chose four biochemistry parameters for analysis cases including Cab (μg/cm^2^), Car (μg/cm^2^), EWT (cm), and LMA (g/cm^2^), which cover a variety of leaf biochemical compositions. Some statistic values for each parameter in the two databases are listed in Table 1. These two experimental databases contain lots of vegetation species, but not enough to represent the variety of GSV, and there is a question about the accuracy of the pigment content in the LOPEX database. Thus, synthetic spectra were simultaneously designed as the third database.

**TABLE 1:** The statistic features for each parameter for two experiment databases.

Statistic feature	Biochemistry parameters			
	Cab (μg/cm^2^)	Car (μg/cm^2^)	EWT (cm)	LMA (g/cm^2^)
Min-value	0.78	0	0.0003	0.0017
Max-value	106.72	28.35	0.053	0.033
Mean-value	41.07	9.55	0.0115	0.0053
Standard deviation	20.56	4.70	0.0060	0.0031

Some studies indicated that these parameter features mostly follow a Gaussian distribution, while for EWT is fitted with lognormal distribution more definitely (Féret et al., 2011). Hereby, distribution information of the four parameters should be considered when generating the synthetic data. It is also noteworthy that the spectra feature of vegetation (including R and T) are affected by numerous biochemical parameters, and some of them are also highly correlated with each other in some special spectral bands. Thus, a synthetic database with compound distribution information of biochemical parameters has been designed to model R and T spectra and then to test the inversion strategy in this study. The synthetic data was synthesized with two parts, i.e., D1 in Table 2, Gaussian distribution considered high correlation among each parameter and D2 of Gaussian distribution. Each part of the synthetic database has a total of 500 spectral samples with 2% Gaussian noise for each band and its statistic features for each parameter were based on the experimental values of the measured database in Table 1.

**TABLE 2:** The *R*^2^/RMSE of FW- and PCA-ANNs models for analyzing four parameters together based on two synthetic databases.

Spectra	FW-ANNs (*R*^2^/RMSE)				PCA-ANNs (*R*^2^/RMSE)			
	Cab	Car	EWT	LMA	Cab	Car	EWT	LMA
	**D1:** *Gaussian distribution considered high correlation among each parameter*							
R	0.46/7.07^1^	0.35/1.62^3^	0.59/0.0019^1^	0.71/0.0011^1^	0.39/6.47^1^	0.40/1.12^1^	0.82/0.0019^3^	0.75/0.0012^3^
T	0.52/6.00^1^	0.48/1.09^2^	0.65/0.0017^4^	0.79/0.001^4^	0.74/8.36^2^	0.70/1.64^2^	0.79/0.0021^3^	0.72/0.0013^3^
R & T	0.76/8.92^2^	0.75/1.67^2^	0.87/0.0019^4^	0.80/9.3e-4^4^	0.76/7.73^2^	0.70/1.58^2^	0.84/0.0022^3^	0.77/0.0013^3^
	**D2:** *Gaussian distribution*							
R	0.88/18.34^1^	0.68/4.36^2^	0.84/0.0027^1^	0.73/0.0026^3^	0.81/14.96^4^	0.91/3.18^1^	0.82/0.0029^4^	0.82/0.0013^4^
T	0.76/21.06^4^	0.71/4.86^3^	0.87/0.0025^1^	0.84/0.0022^1^	0.67/10.48^1^	0.826/3.41^4^	0.90/0.0023^1^	0.89/0.0015^4^
R & T	0.78/27.83^1^	0.71/6.11^4^	0.73/0.0198^4^	0.66/0.0032^1^	/	/	/	/

*In the analysis process, different train functions of ANN models are chosen to optimize the inversion results. The training functions of ANN models include: ^1^Levenberg-Marquardt Algorithm (trainlm), ^2^Bayesian regularization algorithm (trainbr), ^3^Quasi-Newton Algorithm (trainbfg), and ^4^One Step Secant Algorithm (trainoss).*

### The Inversion Strategy of Biochemistry Parameters

In this study, four biochemistry parameters were inverted. The inversion accuracy of them greatly depended on the appropriate inversion strategy because of the performance of algorithms and correlation between biochemistry parameters. Thus, the analysis process in the present investigation is designed as follows: (1) different spectral characteristics (i.e., R, T, and R&T) are considered as input variables of ANNs; (2) four biochemistry parameters are separated as the single input of the ANNs model and then inverted simultaneously; and (3) the inversion process for EWT and LMA is conducted repeatedly in a spectral subset after sensitivity analysis based on R and T spectra. In the abovementioned analysis process, the input variables of ANN were selected using two different characteristic selection methods, described in section “Methods of Spectral Characteristics Selection.” The inversion results were then compared with that using all the spectral characteristics and then the PLS regression (PLSR) method.

**FIGURE 1:**
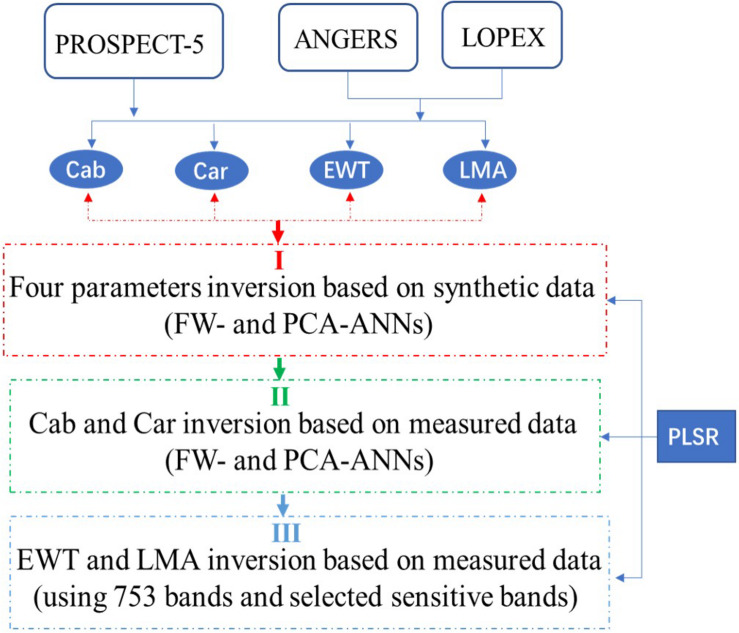
The parameter inversion strategy used in this study. **(I)** Four parameters were inverted based on the synthetic data using FW- and PCA-ANNs; and then **(II)** FW- and PCA-ANN model was utilized to invert Cab and Car based on measured data; **(III)** after resetting the sensitive band range, EWT and LMA were inverted successively.

The parameters EWT and LMA are sensitive with spectral characteristics mostly in the short wavelength infrared (SWIR) range (approximately 1300–2000 nm), which is also correlated with the leaf structure (Ceccato et al., 2002). Thus, the correlation effects must be considered when analyzing EWT and LMA. On this basis, we extended and reset the sensitive band ranges of EWT and LMA (i.e., 750–950, 1300–1700, and 1850–2000 nm) with a total of 753 wavelengths. Then, the abovementioned analysis processes are conducted again. The inversion strategy was presented in Figure 1.

### Artificial Neural Networks

Traditional feed-forward ANNs are used in this study, which consist of three layers (i.e., input, hidden, and output). The ANNs work by learning from the input information *x*_i_, and then freely change and modify the synaptic weights ai of *x*_i_. After summing all these modified neurons, the output values *y*_i_ can be represented by *u*_i_ (Eq. 1) and bias *b*_i_ using a non-linear activation function.

(1) $$u_{i}=\sum_{i=1}^{n}a_{i}x_{i}$$

Where *n* is the input number. The ANNs are trained iteratively to minimize the mean square error (MSE, Eq. 2) between the network outputs and inputs, namely the biochemistry parameter contents and leaf spectral characters respectively in this paper. The four biochemistry parameters can be used as a whole or single dependent variable for the ANN model.

(2) $$MSE=\frac{\sum_{i=1}^{N}{\left(y_{PRE}-y_{MEA}\right)}^{2}}{N}$$

Where *N* is the sample number, and *y*_PRE_ and *y*_MEA_ denote the predicted and measured values, respectively. Many train functions are available for ANNs analysis. We select four commonly used functions, i.e., Levenberg-Marquardt Algorithm (trainlm), Bayesian regularization algorithm (trainbr), Quasi-Newton Algorithm (trainbfg), and One Step Secant Algorithm (trainoss). For medium-scale networks, the trainlm is the fastest training algorithm, and its Jacobian matrix can be divided into several sub-matrixes for large networks, which can overcome the disadvantage of occupying large amounts of memory. The trainbr works as a Bayesian regularization process that updates the weight and bias values based on the L-M algorithm. It minimizes the combination of squared errors and weights which are then determined to produce and optimize a network that generalizes well. The trainbfg needs more storage space, but uses fewer iterations and time per iteration than the other methods when it converges, which is more suitable for small networks. More details about these training functions can be found in the book (Yegnanarayana, 2009) and the documents of software MATLAB 2014b.

In present ANNs, the MSE is 10-5, the hidden size is 10, and the maximum iterations (epochs) are 100. The ANN training process will terminate once each condition is met, namely obtaining an optimal network model. Then, the created networks can be used to validate and test the remaining 20% of data. Additionally, the coefficient of determination (*R*^2^, Eq. 3, $\bar{\text{y}}$ is average of *y*_MEA_) and the root-mean-square error (RMSE) of output layers were implemented to indicate the prediction performance of ANNs. High *R*^2^ and low RMSE indicates the high accuracy of the ANNs model in predicting vegetation biochemistry parameters.

(3) $$R^{2}=1-\frac{\sum {\left(y_{PRE}-y_{MEA}\right)}^{2}}{\sum {\left(y_{PRE}-\bar{y}\right)}^{2}}$$

### Methods of Spectral Characteristics Selection

There are more than 2000 wavelengths in both databases, which indicates a heavy computation for ANNs. Furthermore, most of the spectral characteristics in these wavelengths are correlated with each other, which is unhelpful for the biochemistry parameters analysis. Thus, preselecting the spectral feature is both necessary and helpful in improving the performance of ANNs in the special inversion strategy. In this paper, a FW-based method (Huang and He, 2005) is used to decrease the spectral dimension and acquire sensitive bands for biochemistry parameters. Through this, the inputs of the ANN model change to the reordered spectral characteristics rather than the entire original spectral data.

We assume that the spectral data can be divided into m classes (*j* = 1,2,3…*m*) according to the vegetation species and biochemicals contents in this study. Then, the divergence of the *j*_th_ class is calculated, which presents the significant coefficient of band λ corresponding to class *j*, (η_*j*_(λ), Eq. 5). This coefficient is in a descending sequence according to the sensitivity to the different biochemistry parameters. Subsequently, the FWs are calculated by seeking the band position *p*_re–ranked_ in η_*j*_(λ) according to Eq. 4,

(4) $$w\left(\lambda \right)=\frac{1}{n}\sum_{j=1}^{m}\left(P_{re-ranked}\right)$$

(5) $$\eta_{j}\left(\lambda \right)=\frac{1}{n}\sum_{i=1}^{n}\mu_{i}{\left(E_{\lambda i}\right)}^{2}\left(i=1,2,\ldots ,n\right)$$

where *n* is the number of bands and μ*_i_* represents a divergence ratio of the *i*_th_ band to all bands. Then, the optimal channel combinations are selected for biochemistry parameters analysis by comparing the determination coefficient *R*^2^.

As it is the same as the FW-based method, PCA can decrease the dimension of the spectra by analyzing the internal correlation of the database. By using matrix computation and analysis, the most significant spectral characteristics from the database are extracted by PCA, and instead the linear combinations of the original variables are used as ANNs inputs.

## Results and Comparisons

In this section, we firstly invert the above four parameters follow the inversion strategy described in section “The Inversion Strategy of Biochemistry Parameters” based on synthetic databases (section “Four Biochemistry Parameters Analysis Based on Two Synthetic Databases”). According to the inversion result based on the synthetic database, the parameters were separated or combined as different input forms for the ANN model in sections “FW-ANNs for Cab and Car Analysis With Different Spectral Bands” and “PCA-ANNs for Cab and Car Analysis,” and different numbers of spectral characteristics have been selected from more than 2000 spectral bands by using two kinds of ANNs in both experimental databases. Given the results for EWT and LMA were unsatisfactory, the sensitive analysis has been utilized in section “EWT and LMA Analysis After Sensitive Band Selection and Reordering” to reset a new range for EWT and LMA, and then these were reanalyzed using FW- and PCA-ANNs successively. The PLSR method was conducted as a comparison experiment in this section.

### Four Biochemistry Parameters Analysis Based on Two Synthetic Databases

Based on synthetic databases modeled by PROSPECT-5, these four biochemistry parameters were inverted using FW- and PCA-ANNs method in this section. As mentioned in section “Databases Description,” parameters are highly correlated with each other, which should be an important factor when synthesizing parameters distribution. Thus, a comparison analysis based on the synthetic database without considering the high correlation among each parameter has also been conducted (D2 in Table 2). Compared with using a single parameter as the ANNs input, inverting all of them together produced a higher *R*^2^, especially by combining Cab and Car, and EWT and LMA together respectively (the results about separately inverting them can be seen in Supplementary Table 1). The results in D2 show a higher *R*^2^ but also a higher RMSE, even more so than the standard deviation of parameters, which means the inversion accuracy is highly affected by correlations between each parameter. Maybe this can explain why the combinations of Cab & Car and EWT & LMA as the ANN model inputs respectively could obtain a relatively high *R*^2^. We also find the spectra of R&T can improve the inversion *R*^2^ significantly based on the synthetic database D1, especially when the R or T spectra has poor ability in Cab and Car inversion. However, this result cannot be found in D2.

**FIGURE 2:**
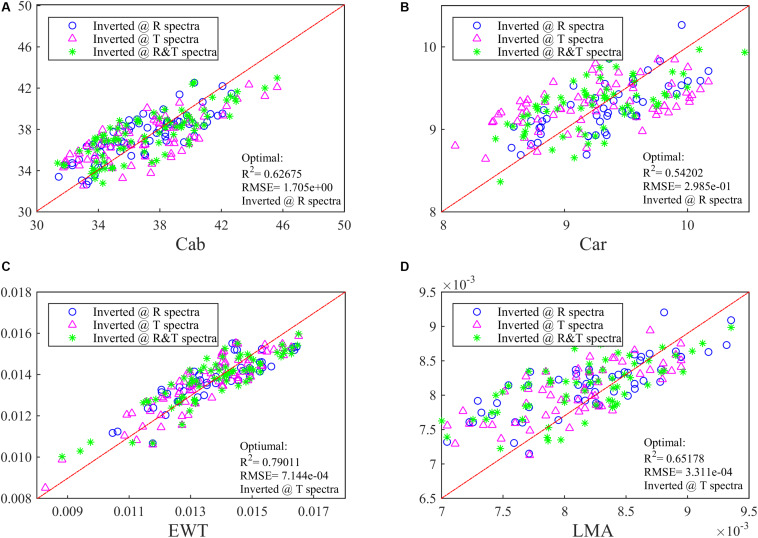
The inversion *R*^2^ for **(A)** Cab, **(B)** Car, **(C)** EWT, and **(D)** LMA in the synthetic database by using PLSR. Different spectra (i.e., R, T, and R&T) are used and the optimal *R*^2^ has been added in the lower right of the figure.

At the same time, the PLSR method has been conducted to invert these four parameters and obtain a relatively low accuracy. The optimal *R*^2^ values for Cab and Car can be indicated with R spectra, while for EWT and LMA the T spectra is a better choice with a *R*^2^ of >0.65 (Figure 2). In Figure 2, combining R and T spectra together is no longer advantageous in biochemistry parameters inversion whose optimal *R*^2^ is even lower than whichever ANN model is being used.

### FW-ANNs for Cab and Car Analysis With Different Spectral Bands

**FIGURE 3:**
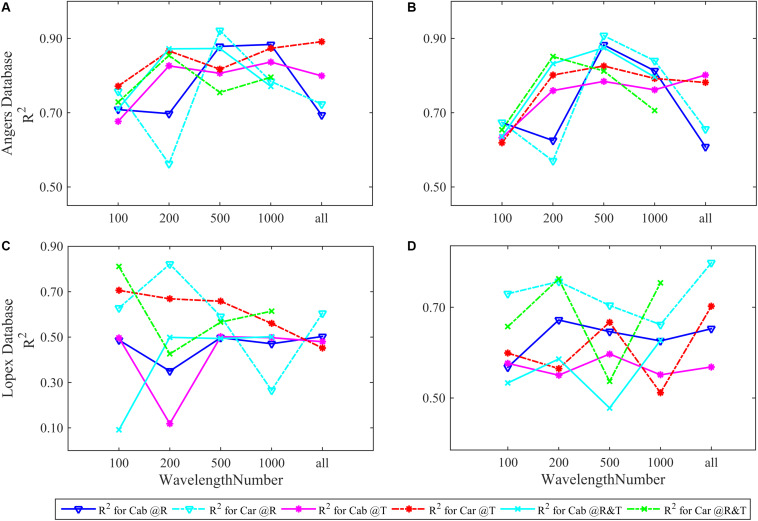
The *R*^2^ of FW-ANNs models for Cab and Car analysis using different numbers of R, T, and R&T spectral bands based on the ANGERS and LOPEX databases. Subpanels **(A)**, **(C)** show the *R*^2^ of separately inverting four parameters and **(B)**, **(D)** is that combining them as a whole of dependent variable for FW-ANNs analysis. The optimal train function of FW-ANN model is trainbr.

Following what has been done in the synthetic database, four parameters are separately and then simultaneously inverted based on experimental data. In this section, we firstly analyzed the model performance for Cab and Car. As for EWT and LMA, the results are unsatisfactory in both databases whether inverting separately or together, which will be reanalyzed in section “EWT and LMA Analysis After Sensitive Band Selection and Reordering” in more detail. The results show that FW-ANN models exhibit a unique ability in parameters inversion by utilizing different number of sensitive bands. These spectral characteristics are reordered in a prior sequence on the basis of the FW values of each wavelength. In this sequence, the first *t* bands have been chosen as the input variables of the ANN model for biochemical analysis (*t* = 100, 200, 500, 1000), and then compared with all spectral bands were used (i.e., *t* = 2051 and 2101 for the ANGERS and LOPEX databases, respectively, in Figure 3).

In the ANGERS database, most ANN models for Cab and Car obtain a *R*^2^ of >0.7 and require fewer than 2051 bands. When all parameters are simultaneously inverted (Figure 3B), the highest *R*^2^ for Cab and Car is obtained with 500 feature bands regardless of the used spectra (R, T, or R&T, the optimal *R*^2^ are listed in Table 3). Being separated as a single dependent variable for ANN models, the required band numbers for the optimal *R*^2^ can be decreased to 200, except for R spectra which require 500 sensitive bands (Figure 3A), with a *R*^2^ of approximately >0.9. Once a high *R*^2^ is obtained, the performance of FW-ANN models gradually degenerate until all bands are utilized. Compared with the ANGERS database, the *R*^2^ values of the LOPEX are generally lower (Table 3). Concretely, improvement of *R*^2^ is obscured when simultaneously inverting all biochemicals, even needing 2101 bands to get a higher *R*^2^. Typically, Car inversion with an R spectra can get an *R*^2^ approximately 0.82 with all 2101 bands, which is almost the same with that using 200 bands (Figure 3D). The model *R*^2^ with T or R&T spectra for Car inversion can be close to 0.8 and the needed bands numbers are just 100 when completely separating them. However, the model performance tend to be lower than 0.6 when bands are increased (Figure 3C). Being different from the results in section “Four Biochemistry Parameters Analysis Based on Two Synthetic Databases,” combining Cab and Car together or using the R&T spectra is no longer enough to improve the inversion accuracy of ANN models.

**TABLE 3:** The optimal *R*^2^ and their corresponding RMSEs for the FW-ANN models selected from five different bands.

Model accuracy	Separated						Together					
	Cab			Car			Cab			Car		
	R	T	R&T	R	T	R&T	R	T	R&T	R	T	R&T
	*ANGERS*											
*R*^2^	0.88	0.83	0.87	0.92	0.89	0.85	0.88	0.80	0.87	0.91	0.83	0.85
RMSE	6.12	7.62	8.00	1.26	1.21	2.31	6.94	8.97	8.78	1.53	1.76	2.49
	*LOPEX*											
R^2^	0.50	0.50	0.50	0.82	0.71	0.81	0.67	0.60	0.63	0.80	0.70	0.76
RMSE	3.08	3.06	3.36	1.03	1.13	0.87	8.19	9.78	6.93	1.00	1.48	0.95

*In the analysis process of Cab and Car, R, T, and R&T spectra are used for two measured databases.*

### PCA-ANNs for Cab and Car Analysis

**FIGURE 4:**
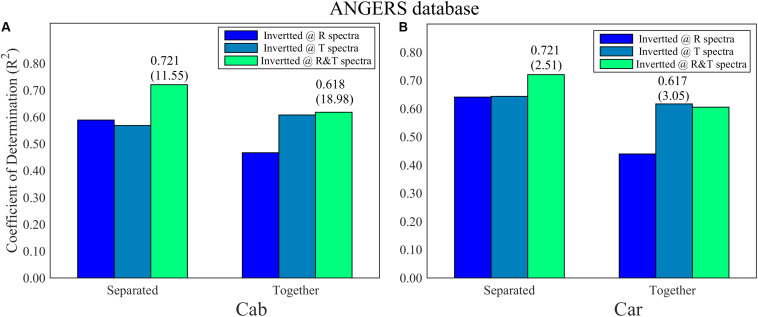
The *R*^2^ and its RMSE (in bracket) for **(A)** Cab and **(B)** Car analysis based on PCA-ANN models in ANGERS. The different colors of the histogram indicate the inverting strategy using R, T, and R&T spectra, respectively. The optimal train function in this section is trainbr.

**FIGURE 5:**
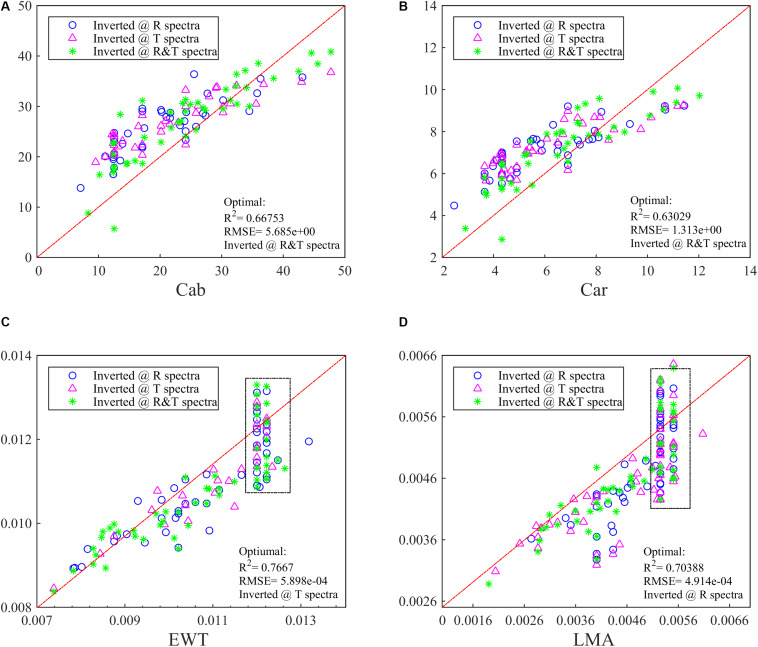
The inversion *R*^2^ for **(A)** Cab, **(B)** Car, **(C)** EWT, and **(D)** LMA in ANGERS by using PLSR. The optimal *R*^2^ with different spectra has been added in the lower right of the figure.

For the ANGERS database, completely separating the four parameters as the single dependent variables for PCA-ANN models generally achieves a higher *R*^2^ than using them as whole dependent variables for PCA-ANNs, except for that using T spectra to analyze Cab (Figure 4A). For both Cab and Car, the PCA-ANN models obtain almost similarly high *R*^2^ values with R or T spectra. The model *R*^2^ can even be increased to 0.72 with R&T spectra when separately inverting them. However, the results for EWT and LMA are unsatisfactory. For the LOPEX database, most *R*^2^ values for four parameters are so low (being not more than 0.4 which can be found in Supplementary Figure 1) that they can be considered as a failure inversion. Besides, by combining R&T spectra, PLSR method can indicate the Cab and Car with an optimal *R*^2^ in ANGERS, which is consistent with using PCA-ANNs (Figures 5A,B). However, the *R*^2^ values are slightly lower than in PCA-ANN models which has an *R*^2^ of > 0.7.

Compared with FW-ANNs, the optimal *R*^2^ values of PCA-ANNs are lower and are generally achieved by combining R and T spectra together. This weakness is greatly apparent on the LOPEX database (in Supplementary Material 2). However, PCA-ANNs use fewer than four PCs for Cab and Car analysis but still obtain a *R*^2^ of >0.7, which has a more efficient analysis process.

### EWT and LMA Analysis After Sensitive Band Selection and Reordering

In the entire spectral range, satisfactory results haven’t been achieved whichever training function of ANN is being used. Hence, the sensitive analysis among biochemistry parameters was conducted in this section to find new sensitive spectral bands for EWT and LMA. Evidently, the effected spectral bands for EWT are mainly located in the SWIR range, which are mixed with other parameters, including LMA which exhibits a close correlation with another spectral range in NIR. Thus, obtaining sensitive ranges covering the NIR and SWIR is both necessary and effective for improving the analysis process of EWT and LMA. In this section, the sensitive bands include 750–950, 1300–1700, and 1850–2000 nm with a total of 753 wavelengths for ANN models. As the compare experiment, all of these 753 bands are used to invert EWT and LMA, and then FW- and PCA-ANNs are used to invert them, as was seen in the Cab and Car in sections “FW-ANNs for Cab and Car Analysis With Different Spectral Bands” and “PCA-ANNs for Cab and Car Analysis.”

#### Analyzing EWT and LMA With 753 Wavelengths by Using ANNs

For the ANGERS database, the T-based ANNs seem to be more useful and efficient for EWT and LMA analysis than using R or a combined R&T spectra with 753 wavelengths (Table 4). And the ANN models of the EWT and LMA analysis can be evidently improved by combining the R and T spectra together. When integrating these 753 picked-up spectral characteristics, the *R*^2^ of ANN models can be considerably increased to >0.55, which is significantly higher than that without resetting sensitive ranges (as low as 0.2–0.4) despite using R, T, or R&T spectra. In contrast to PCA-ANNs for Cab and Car analysis in Section “FW-ANNs for Cab and Car Analysis With Different Spectral Bands,” EWT and LMA analyzed together have a better result than being separated, especially using R or T spectra. The only exception occurs when separately analyzing LMA using R&T spectra, which obtains an *R*^2^ of approximately 0.53; this result is better than analyzing EWT and LMA together (Table 4).

**TABLE 4:** The optimal *R*^2^ of ANN models for EWT and LMA analysis by using 753 R, T, and R&T spectral bands respectively.

	Separated		Together		Separated		Together	
Spectra	EWT	LMA	EWT	LMA	EWT	LMA	EWT	LMA
	*ANGERS*				*LOPEX*			
R	0.4444^3^	0.4973^4^	0.5465^3^	0.5142^3^	0.2342^4^	0.3815^3^	0.3982^3^	0.4360^4^
T	0.5526^1^	0.5533^3^	0.5751^1^	0.5537^1^	0.2487^3^	0.2192^4^	0.3362^4^	0.1901^4^
R & T	0.4947^4^	0.5292^4^	0.5109^1^	0.5062^4^	0.4263^3^	0.3146^4^	0.4263^3^	0.4531^4^

*In the analysis process, different train functions of ANNs model are used (listed in Table 2). The corresponding RMSE can be found in Supplementary Material 3. The training functions of ANN models include: ^1^Levenberg-Marquardt Algorithm (trainlm), ^3^Quasi-Newton Algorithm (trainbfg), and ^4^One Step Secant Algorithm (trainoss).*

Compared with the results in the ANGER database, the analysis results based on the LOPEX database are not entirely satisfactory for estimating the efficiency of sensitive bands selection and reordering, which can almost be considered as a failure analysis process. This lower result is consistent with the results obtained by PCA-ANN analysis for the LOPEX database. However, some *R*^2^ values increase to > 0.4 when simultaneously analyzing EWT and LMA, such as in the R&T-based model, which is higher than using separated parameters as the dependent variables of ANNs models.

#### Analyzing EWT and LMA Based on FW- and PCA-ANNs

In consideration of the poor inversion ability with all spectral bands, the FW-ANNs were utilized to select a different number of reordered sensitive bands (include the first 20, 50, 100, 200, and 500 bands) to analyse EWT and LMA in the new spectral range. PCA-ANN models with all 753 bands were then operated successively. Certainly, the PLSR method was also conducted to compare their ability in EWT and LMA inversion.

**FIGURE 6:**
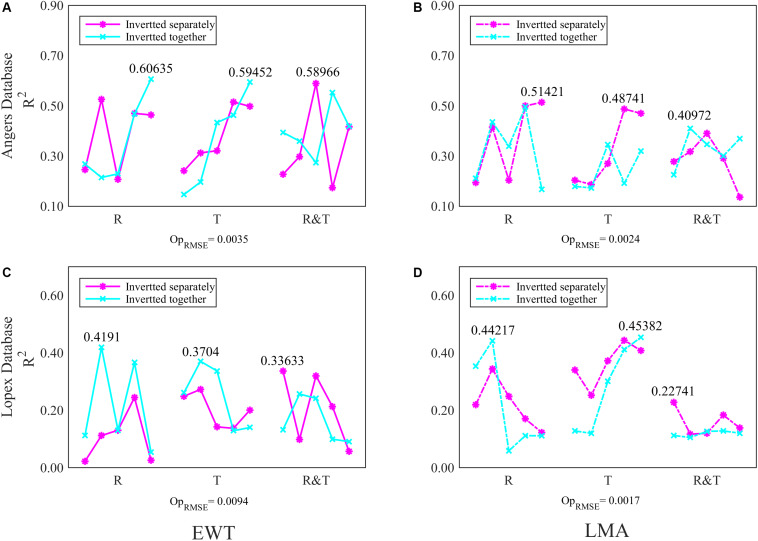
The *R*^2^ of FW-ANN models for **(A)**, **(B)** EWT and **(C)**, **(D)** LMA analysis in two databases. The first 20, 50, 100, 200, and 500 (five scatters in each spectral group) spectral bands of R, T, and R&T were chosen for analysis models respectively. The optimal train function is trainbfg.

In the ANGERS database, the model *R*^2^ of EWT based on the R or T spectra is improved to approximately 0.6 (Figure 6A) as the input variables increase to 500. However, FW-ANNs models with different spectra bands are inefficient in improving the analysis results of LMA; even most inversion strategies obtain a lower *R*^2^ value than using all 753 bands with ANNs (Table 4 and Figure 6B). Moreover, in the ANGERS database, the *R*^2^ for EWT analysis with 100 R&T bands is approximately 0.6 when separating it with LMA, while the *R*^2^ is just approximately 0.5 even though all 753 bands are used for the ANNs models.

Generally, the *R*^2^ values for the EWT and LMA analysis of FW-ANNs model in the LOPEX databases are unsatisfactory, but some evident improvements are still observed (Figure 6C). For instance, together with EWT, model *R*^2^ of LMA with the first 50 R spectral bands is almost the same with using 753 R&T bands in Table 4. Moreover, the FW-ANNs model has improved the performance of T spectra on EWT and LMA inversion, which can even obtain an *R*^2^ of >0.45 (Figure 6D).

**FIGURE 7:**
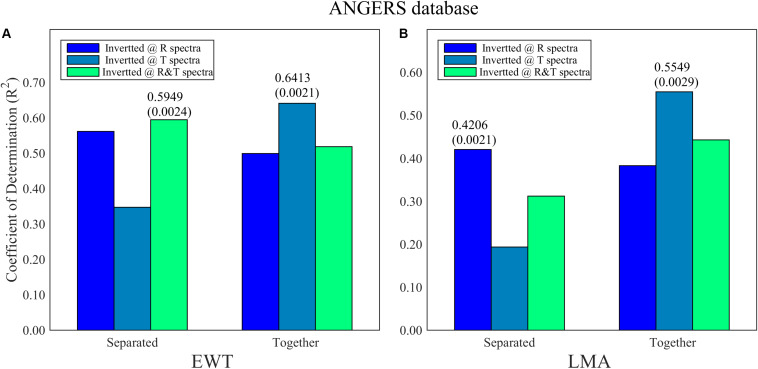
The optimal *R*^2^ and its corresponding RMSE (in bracket) of PCA-ANN models for **(A)** EWT and **(B)** LMA analysis being separated and together in ANGERS. A total of 753 bands reset and reordered from 2051 wavelengths are used. The PCs of analysis model are less than four.

The improvements of the model are evident when resetting sensitive ranges, especially for the ANGERS database based on PCA-ANNs. Compared with the results in Figure 7 and Table 2, the reset range contains a considerable amount of useful information about EWT and LMA, thereby making a big difference in inverting these two biochemistry parameters whatever using R, T, or R&T spectra. With regard to separating or integrating EWT and LMA by using PCA-ANN, the results don’t show a coinciding discipline, but the T spectra greatly contributes to the EWT and LMA analysis, especially when EWT and LMA are the dependent variables of PCA-ANN models simultaneously (Figure 7, 0.64 for EWT and 0.56 for LMA).

By using the PCA method among the reset 753 wavelengths, separately analyzing EWT based on R and R&T spectra has been ameliorated better (from low than 0.5 to 0.59, Table 4 and Figure 7A). And the *R*^2^ can even be increased to 0.64 by integrating LMA together as the inputs of T-based ANN models. For LMA analysis based on PCA-ANNs, satisfactory *R*^2^ are obtained using T spectra integrating EWT together, which is consistent with that using ANNs analysis with all 753 bands in section “Analyzing EWT and LMA With 753 Wavelengths by Using ANNs” (Table 4). Thus, it is a good analysis strategy to invert EWT and LMA together. Similar to FW-ANNs, the *R*^2^ of EWT models based on PCA-ANNS in the ANGERS database are relatively higher than only using ANNs without selecting model variables, either using R, T, or R&T spectra. This result indicates that the band-selection-based ANNs in this paper can achieve an efficient improvement for EWT inversion. While for LMA, it is more difficult compared to any other parameters regardless of which band selection method being used. The results for the LOPEX database were presented in Supplementary Figure 2.

On the contrary, PLSR can obtain a great improvement in EWT and LMA analysis, especially for LMA, as its *R*^2^ can be increased from less than 0.6 to 0.7 (Figure 5D). But we should note that some inverted values of EWT and LMA with PLSR correspond to many different real values, marked with a black dotted box in Figures 5C,D, which means the PLSR has the overfitting problem.

## Discussion

### Factors Affected the Model Predictability

The different collection conditions of spectra, including years, sites, and vegetation species, are important factors for the universality of inversion algorithms in practice (Li et al., 2010). In the present study, the spectra in two measured databases were collected by different institutions, and each of them contained fewer than 50 vegetation species. The data from the LOPEX database contained only 64 available spectra, which was a limited sample size for the ANN model training and construction. Moreover, the accuracy of the parameters in the LOPEX database was questionable according to the collection institution report. These two factors would contribute to the predictability of the ANN model for LOPEX, thus, it had a poorer inversion accuracy than the ANGERS database. The effect of these external factors, certainly including that from the surrounding environment, on spectral characteristics can be highlighted by bands selection to accurately analyze biochemistry parameters. More than 2000 spectral bands are present for both the ANGERS and LOPEX database, which is a huge number of characteristics for biochemistry parameter inversion. More useless variables considerably contribute to the computation for the ANN model and increase the probability of the overlearning problem, like overfitting in regression analysis (i.e., PLSR). Fundamentally, most of these spectral bands are unhelpful for inversion improvement, and even obstruct ANN analysis, thereby leading to the decrease of models’ *R*^2^. Thus, FW analysis and PCA have been conducted before the operation of the ANN model to considerably improve the analysis process. In this study, FW- and PCA-ANNs perform a divergent ability on biochemistry parameter inversion with two independent databases. Compared with applying PCA, databases have been divided into different classes according to the above-mentioned factors, such as vegetation species, biochemistry contents, and sampling sites before calculating the FWs of each wavelength to reduce correlations and divergences between different classes, as well as those between mixed spectral bands and each other. Thus, FW-ANN models exhibit gratifying results.

Different training functions presented diversiform performances in biochemistry parameter inversion because of their optimization ability, whether using the FW- or PCA-ANN model in the synthetic database (Table 2). For two measured databases, the optimal training function of the inversion model for Cab and Car was trainbr, and was trainbfg for EWT and LMA. There were some complex correlations between these external factors, which have not been found explicitly, making it difficult to generalize the inversion model with special training function. The present study is the preliminary research of this inversion strategy, and a more detailed study may follow up in the future, which could consider more correlated factors affecting the ANN model predictability for vegetation parameters.

### Spectral Property in Special Band for Each Parameter

The inversion performance greatly depends on the combined forms of spectral properties, such as R only, T only, and R&T, considering the R and T spectral response to biochemistry parameters in different band ranges. A constant range is insufficient to accurately analyze some of the biochemistry parameters. Thus, considerable sensitive band ranges based on R and T properties should be picked up against special parameters or combined parameters. This phenomenon is evident in this study, especially for EWT and LMA. The combined form with considerable spectral properties (R&T) is the optimal input of PCA-ANNs for Cab and Car analysis (Figure 4), but not when using FW-ANNs, which achieves a high *R*^2^ with few hundreds of R or T bands alone (Figure 3). Both EWT and LMA considerably contribute to the R spectra in the SWIR range. However, it is difficult to precisely invert any of them by SWIR range alone, as the other ranges sensitive to EWT or LMA are still needed. The NIR is a good choice, as it is greatly influenced by LMA but not highly important for Cab and Car. Thus, analyzing and then choosing some suitable bands from R and T spectra, such as the FW- and PCA-based selection conducted on two dependent databases in this study, is an efficient inversion strategy for special biochemistry parameters before using ANNs models.

As mentioned earlier, the special bands of EWT and LMA are almost located in NIR and SWIR, which have a poor relationship with Cab and Car. The Cab and Car are mainly correlated with visible bands. When inverting these four parameters together in the entire spectral range, this outstanding feature may be overwhelmed by redundant correlations even though the FW and PCA method are conducted to select the spectral characteristics before ANN analysis. Instead, by separating them as the single dependent variables of ANN models, the optimal results for Cab and Car have been obtained whether using FW-ANNs or PCA-ANNs. In contrast, the LMA mainly dominates the R spectral range in NIR and SWIR which is also the main sensitive range of EWT. Thus, the inversion accuracy of EWT and LMA is mixed and greatly depends on whether both of them are being analyzed simultaneously. Moreover, the T spectra selected by PCA or in all 753 wavelengths outperform any of the R and R&T spectra in inverting EWT and LMA together. Light penetrating into the leaf can be partially absorbed by biochemicals, and the remaining light transmitted out from the leaf is called the T spectra. Hypothetically, the T spectra related to the amount of radiation absorbed by EWT and LMA can obtain a better analysis than relying on the R spectra (Richardson et al., 2010).

### Spectra Simulation and Fusing

The inversion strategy in this study is always desired to be tested in more and more measured databases, which can offer valuable guidance in vegetation parameter inversion. However, there are limited measured databases after all, and some unknown factors during the measurement may decrease the database availability. Meanwhile, the different measurement conditions, such as the spectral resolution or numbers of parameters, make data combination or fusing for parameter inversion difficult. An optimal inversion model can be established by simultaneously using R and T spectra, such as the results for Cab and Car analysis in Figure 3, more so than simply fusing R plus T (Du et al., 2017), such as the data assimilation of R and T spectra, even when including more spectral properties, i.e., the fluorescence of chlorophyll. This has been shown to be a well-rounded idea for biochemistry parameter analysis (Grace et al., 2010; Stöckli et al., 2015; Li et al., 2017). Moreover, the PROSPECT, Markov-Chain Canopy Reflectance Model, or the other physical models of the transformation of radiation in vegetation leaf and canopy levels could be another potential topic in accurately and efficiently analyzing biochemistry parameters (Kuusk, 2001; Féret et al., 2017).

## Conclusion

For the ANGERS and LOPEX databases, the selected spectral properties of FW and PCA include prior and dominated information about the four biochemicals. The FW-and PCA-ANN models have efficiently improved the inversion results by selecting fewer but optimal spectral variables than using ANN analysis alone. Finding these feature ranges and then judging whether all or some of them should be inverted together is an efficient analysis strategy before conducting ANN models because of the mixture and complexity of each parameter in different sensitive ranges (e.g., being together is optimal for EWT and LMA, while being separated is fine for Cab and Car in this study). This condition is an interesting and valuable topic worthy of further study.

## Data Availability Statement

All datasets generated for this study are included in the article/Supplementary Material.

## Author Contributions

LD and WG conceived and designed the experiments. LD, SS, and JS performed the experiments. SS and LD analyzed the data. JY and JS contributed the materials and analysis tools. LD wrote the manuscript.

## Conflict of Interest

The authors declare that the research was conducted in the absence of any commercial or financial relationships that could be construed as a potential conflict of interest.
