# Supplementary material for: Leaf Biochemistry Parameters Estimation of Vegetation Using the Appropriate Inversion Strategy
Source: Front Plant Sci. 2020 May 20;11:533. doi: 10.3389/fpls.2020.00533 (PMC7326141; doi:10.3389/fpls.2020.00533)
Supplement: Supplementary file 1 [file Table_1.DOCX]

**Support: Table 1**

The *R*^2^/RMSE of FW- and PCA-ANNs models by separately inverting these four parameters based on synthetic database.

|  | FW-ANNs (*R*^2^/RMSE) | | | | PCA-ANNs (*R*^2^/RMSE) | | | | |
| --- | --- | --- | --- | --- | --- | --- | --- | --- | --- |
|  | Cab | Car | EWT | LMA | | Cab | Car | EWT | LMA |
| **D1:** *Gaussian distribution considered high correlation among each parameter* | | | | | | | | | |
| R | 0.41/6.99^2^ | 0.30/1.49^1^ | 0.55/0.0013^3^ | 0.70/0.0012^4^ | | 0.39/6.47^1^ | 0.37/1.21^3^ | 0.40/0.0022^1^ | 0.21/0.0016^1^ |
| T | 0.45/5.81^3^ | 0.47/1.12^3^ | 0.47/0.0017^2^ | 0.77/0.0011^1^ | | 0.51/5.13^1^ | 0.39/0.95^1^ | 0.58/0.0015^2^ | 0.54/0.001^1^ |
| R & T | 0.54/6.26^2^ | 0.71/1.57^3^ | 0.65/0.0014^1^ | 0.60/7.62e-4^2^ | | 0.56/5.24^3^ | 0.67/1.07^4^ |  |  |
| **D2:** *Gaussian distribution* | | | | | | | | | |
| R | 0.57/21.70^3^ | 0.57/2.70^1^ | 0.70/0.0028^4^ | 0.66/0.0024^4^ | | 0.56/11.11^2^ | / | 0.69/0.0025^3^ | 0.30/0.0019^4^ |
| T | 0.56/20.65^2^ | 0.61/4.43^3^ | 0.66/0.0028^1^ | 0.79/0.002^1^ | | 0.53/9.41^2^ | 0.72/2.36^4^ | 0.49/0.002^4^ | 0.51/0.001^3^ |
| R & T | 0.74/26.00^3^ | 0.71/6.11^3^ | 0.59/0.026^2^ | 0.66/0.0032^3^ | | / | / | / | / |
