# Supplementary material for: Leaf Biochemistry Parameters Estimation of Vegetation Using the Appropriate Inversion Strategy
Source: Front Plant Sci. 2020 May 20;11:533. doi: 10.3389/fpls.2020.00533 (PMC7326141; doi:10.3389/fpls.2020.00533)
Supplement: Supplementary file 2 [file Table_2.DOCX]

**Support: Table 2**

The *R^2^* of PCA-ANNs models for four parameters analysis using R, T and R&T spectral bands for two databases. In the analysis process, different train functions of ANN models are chosen to optimize the inversion results.

|  | Separated | | | | Together | | | | |
| --- | --- | --- | --- | --- | --- | --- | --- | --- | --- |
|  | Cab | Car | EWT | LMA | | Cab | Car | EWT | LMA |
| *ANGERS* | | | | | | | | | |
| R | 0.5889^1^ | 0.7269^2^ | 0.5161^4^ | 0.2752^3^ | | 0.6421^4^ | 0.5548^2^ | 0.2480^4^ | 0.2040^4^ |
| T | 0.6393^3^ | 0.6437^1^ | 0.4293^3^ | 0.2457^3^ | | 0.6078^1^ | 0.6168^1^ | 0.4130^3^ | 0.2262^3^ |
| R & T | 0.7209^1^ | 0.7207^1^ | 0.4592^3^ | 0.3455^3^ | | 0.6181^1^ | 0.6052^1^ | 0.2055^4^ | 0.2306^2^ |
| *LOPEX* | | | | | | | | | |
| R | 0.2887^2^ | 0.2204^2^ | 0.1970^4^ | 0.3298^1^ | | 0.2640^2^ | 0.3980^2^ | 0.3261^2^ | 0.4818^2^ |
| T | 0.1904^4^ | 0.1079^2^ | 0.2163^2^ | 0.3354^2^ | | 0.2478^2^ | 0.2193^3^ | 0.1958^2^ | 0.2192^3^ |
| R & T | 0.2309^2^ | 0.2642^2^ | 0.2305^1^ | 0.1775^2,3^ | | 0.3471^2^ | 0.3263^2^ | 0.45^2^ | 0.3847^3^ |

**Support: Table 3**

The corresponding RMSE for **Support Table 2**.

|  | Separated | | | | Together | | | | |
| --- | --- | --- | --- | --- | --- | --- | --- | --- | --- |
|  | Cab | Car | EWT | LMA | | Cab | Car | EWT | LMA |
| *ANGERS* | | | | | | | | | |
| R | 11.4914^1^ | 2.5832^2^ | 0.0028^4^ | 0.0026^3^ | | 12.2483^4^ | 3.4042^2^ | 0.0032^4^ | 0.2040^4^ |
| T | 14.4435^3^ | 2.6857^1^ | 0.0029^3^ | 0.0025^3^ | | 14.0476^1^ | 3.0534^1^ | 0.0048^3^ | 0.0053^3^ |
| R & T | 11.5458^1^ | 2.5077^1^ | 0.0028^3^ | 0.0021^3^ | | 18.9757^1^ | 4.3117^1^ | 0.0033^4^ | 0.0051^2^ |
| *LOPEX* | | | | | | | | | |
| R | 6.8514^2^ | 1.8326^2^ | 0.0033^4^ | 0.0019^1^ | | 26.3399^2^ | 7.1892^2^ | 0.0048^2^ | 0.0018^2^ |
| T | 26.7758^4^ | 2.9630^2^ | 0.0020^2^ | 0.0024^2^ | | 8.0132^2^ | 2.6911^3^ | 0.0041^2^ | 0.0018^3^ |
| R & T | 8.0035^2^ | 1.9895^2^ | 0.0025^1^ | 0.0038^2,3^ | | 8.0468^2^ | 2.5072^2^ | 0.0057^2^ | 0.0026^3^ |
