# Supplementary material for: Leaf Biochemistry Parameters Estimation of Vegetation Using the Appropriate Inversion Strategy
Source: Front Plant Sci. 2020 May 20;11:533. doi: 10.3389/fpls.2020.00533 (PMC7326141; doi:10.3389/fpls.2020.00533)
Supplement: Supplementary file 3 [file Data_Sheet_1.docx]

**Support: Table 4**

The corresponding RMSE for Table 4 in manuscript.

|  | Separated | | Together | | Separated | | Together | |  |
| --- | --- | --- | --- | --- | --- | --- | --- | --- | --- |
| *ANGERS* | | | | | *LOPEX* | | | | |
|  | EWT | LMA | EWT | LMA | EWT | LMA | EWT | LMA |  |
| R | 0.0034^3^ | 0.0022^4^ | 0.0048^3^ | 0.0014^3^ | 0.0044^4^ | 0.0031^3^ | 0.0035^3^ | 0.0043^4^ |  |
| T | 0.0022^1^ | 0.0021^3^ | 0.0019^1^ | 0.0019^1^ | 0.0029^3^ | 0.0035^4^ | 0.0030^4^ | 0.0096^4^ |  |
| R & T | 0.0034^4^ | 0.0021^4^ | 0.0018^1^ | 0.0034^4^ | 0.0022^3^ | 0.0023^4^ | 0.0024^3^ | 0.0042^4^ |  |
