# Supplementary material for: Leaf Biochemistry Parameters Estimation of Vegetation Using the Appropriate Inversion Strategy
Source: Front Plant Sci. 2020 May 20;11:533. doi: 10.3389/fpls.2020.00533 (PMC7326141; doi:10.3389/fpls.2020.00533)
Supplement: Supplementary file 4 [file Data_Sheet_2.docx]

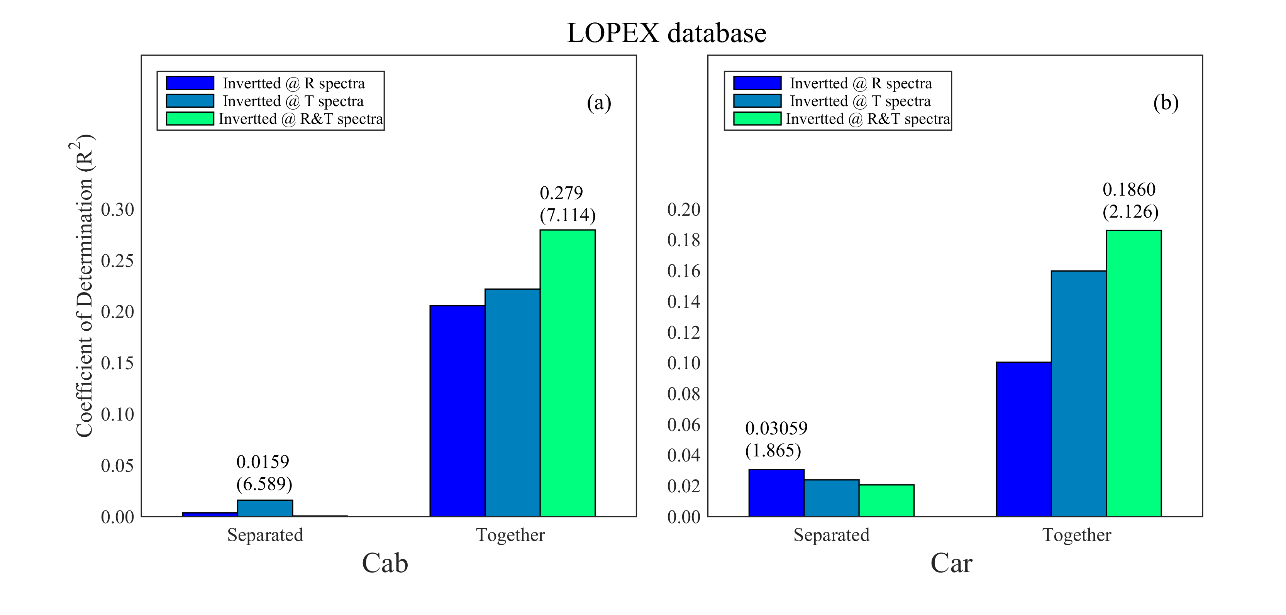
**Surpport: Figure 1**

**Fig. 1.** The R2 and its RMSE (in bracket) for (a) Cab and (b) Car analysis based on PCA-ANNs models in LOPEX. The different colors of the histogram indicate the inverting strategy using R, T and R&T spectra, respectively. The optimal train function in this section is trainbr.

**Surpport: Figure 2**


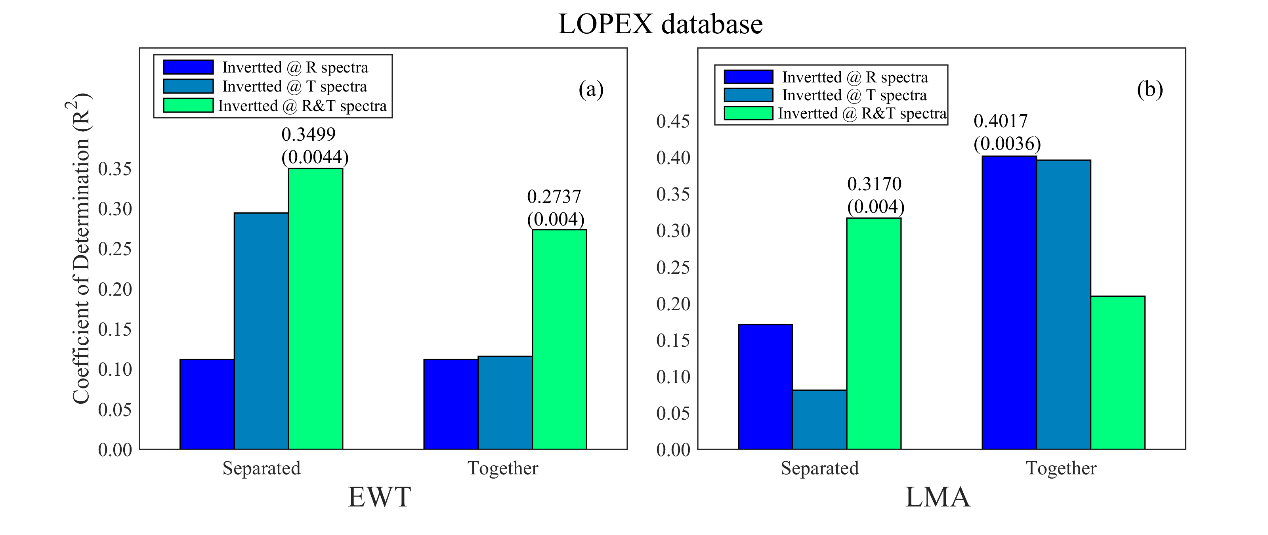
**Fig. 2.** Optimal *R*^2^ and its corresponding RMSE of PCA-ANN models for EWT and LMA analysis being separated and together in LOPEX. A total of 753 bands reset and reordered from 2051 wavelengths are used.
